# Supplementary material for: Intense attosecond pulses carrying orbital angular momentum using laser plasma interactions
Source: Nat Commun. 2019 Dec 5;10:5554. doi: 10.1038/s41467-019-13357-1 (PMC6895158; doi:10.1038/s41467-019-13357-1)
Supplement: Supplementary file 1 — Supplementary Information [file 41467_2019_13357_MOESM1_ESM.pdf]

Supplementary Information for "Intense attosecond pulses carrying orbital angular momentum using laser plasma interactions"

J. W. Wang,[1,*] M. Zepf,[2,3,†] and S. G. Rykovanov[2,4,‡]

[1]*State Key Laboratory of High Field Laser Physics, Shanghai Institute of Optics and Fine Mechanics, Chinese Academy of Sciences, Shanghai 201800, China*

[2]*Helmholtz Institute Jena, Fröbelstieg 3, 07743 Jena, Germany*

[3]*Institut für Optik und Quantenelektronik, Friedrich-Schiller-Universität Jena, Max-Wien-Platz 1, 07743 Jena, Germany*

[4]*Center for Computational and Data-Intensive Science and Engineering, Skolkovo Institute of Science and Technology, Moscow 121205, Russia*

[*] Electronic address: wangjw@siom.ac.cn

[†] Electronic address: m.zepf@uni-jena.de

[‡] Electronic address: S.Rykovanov@skoltech.ru

Supplementary Note 1: The polarization and handedness of the generated harmonics field.

The polarization and handedness of the generated harmonics field are checked by calculating the Stokes parameters of the reflected harmonics field. Since the reflected field is now propagating to $-z$ direction, the fitting coordinate system should be changed to {-**x**, **y**, -**z**} and therefore $\mathbf{E_{-x}} = -\mathbf{E_x}$. Here we take the second order harmonic for example. Its Stokes parameters read

$$S = \begin{Bmatrix} S_0 \\ S_1 \\ S_2 \\ S_3 \end{Bmatrix} = \begin{Bmatrix} E_x E_x^* + E_y E_y^* \\ E_x E_x^* - E_y E_y^* \\ -E_x E_y^* - E_y E_x^* \\ i(-E_x E_y^* + E_y E_x^*) \end{Bmatrix} = I_2 \begin{Bmatrix} 1.000 \\ 0.018 \\ 0.014 \\ 0.999 \end{Bmatrix} \cong I_2 \begin{Bmatrix} 1 \\ 0 \\ 0 \\ 1 \end{Bmatrix}, \tag{1}$$

where I_2 is the relative intensity of the second order harmonic, and E_x, E_y are the electric fields of the second order harmonic in the coordinate system {**x**, **y**, **z**}, respectively. The Stokes parameters clearly show that the second order harmonic is almost circularly polarized and right-handed (as defined from the point of view of the observer). Since the harmonics propagate to $-z$ direction while the left-handed CP driving laser propagates to $+z$ direction, the spin angular momentum of the emitted photon in the harmonics is the same as that of the incident photon.

Supplementary Note 2: Harmonic spectrum for the pre-denting case in the 3D simulation.

The intensity of the harmonic spectrum rolls off according to the power law $1/n^5$ for the few-cycle driving laser pulse.

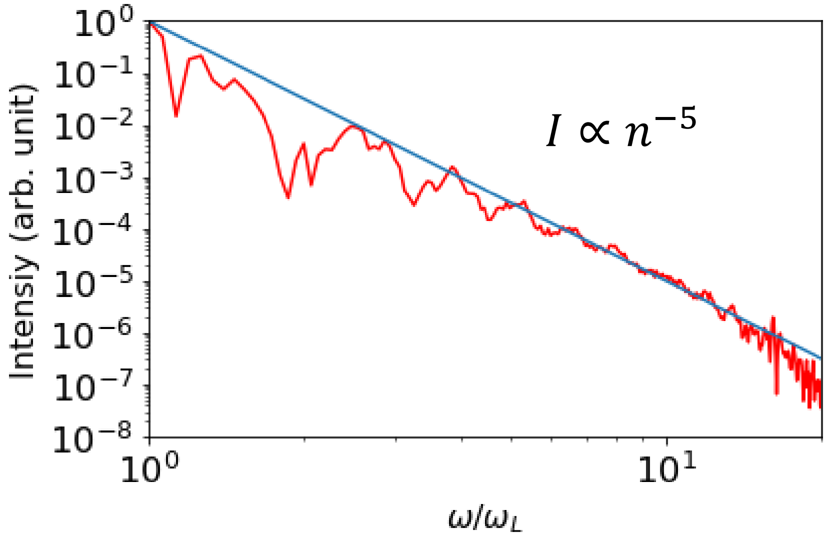

Supplementary Figure 1: Harmonic spectrum for the pre-denting case in the 3D simulation. The laser intensity $a_0 = 2.8$, laser spot radius $w = 4\lambda_\mathrm{L}$ and laser duration $T_0 = 4\tau$. The target is pre-dented. The resolution in the 3D simultion is dx=dy=0.02λ_L, dz=0.01λ_L.

Supplementary Note 3: 2D simulation results for the pre-denting case.

To check the high-order harmonics of the pre-denting case, we carried 2D simulations with a higher resolution dy=dz=0.005λ_{L}. From Supplementary Fig. 2 one can find that up to 30th harmonic is clearly observed. And an attosecond pulse with a duration of 240 as can be obtained by filtering out the low orders ($n \leq 15$) harmonics.

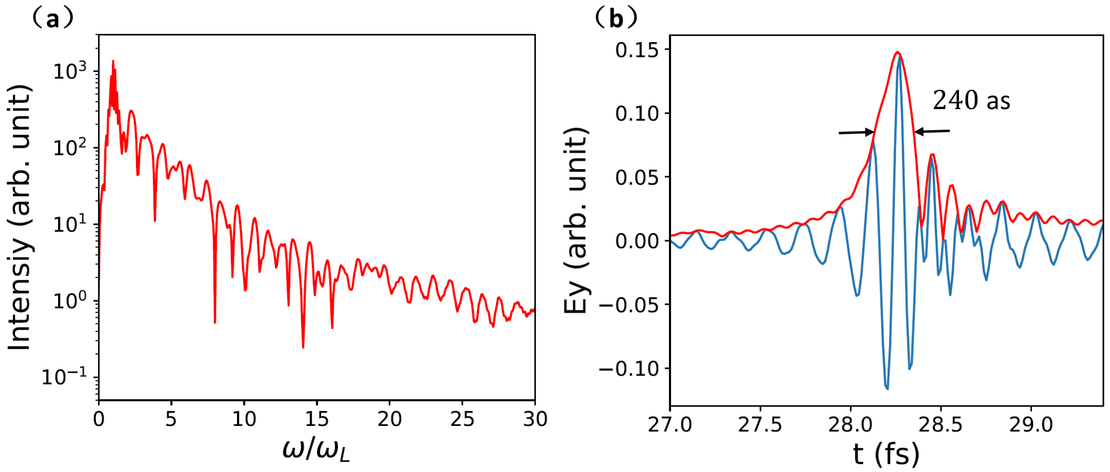

Supplementary Figure 2: 2D simulation results for the pre-denting case. (a) Spectrum of the harmonics. **(b)** Temporal shape of the attosecond pulse by filtering out the low orders ($n \leq 15$) harmonics. The laser intensity $a_0 = 2.8$, laser spot radius $w = 4\lambda_{\mathrm{L}}$ and laser duration $T_0 = 4\tau$. The target is pre-dented. The resolution in the 2D simultion is dy=dz=0.005λ_{L}.

Supplementary Note 4.

We note that when we were submitting our manuscript, a paper appeared in [1] which also discussed SAM to OAM conversion in the relativistic regime. In contrast to our paper it identifies the longitudinal electric field as the driver of the plasma surface oscillation. In fact, the contribution of longitudinal field to the driving force is at least one order of magnitude weaker than that of the transverse field. Therefore, target denting and the subsequent azimuthal dependence of the oscillating force term are the keys to the harmonic generation process and therefor for the SAM-to-OAM conversion in the present scheme.

Supplementary Reference

[1] Li, S. et al. Spin-to-Orbital Conversion of Angular Momentum in Harmonic Generation Driven by Intense Circularly Polarized Beam. Preprint at https://arxiv.org/abs/1812.10255 (2018).
